# From a Cup of Tea to Cardiovascular Care: Vascular Mechanisms of Action

**DOI:** 10.3390/life14091168

**Published:** 2024-09-15

**Authors:** Marios Sagris, Panayotis K. Vlachakis, Spyridon Simantiris, Panagiotis Theofilis, Maria Gerogianni, Paschalis Karakasis, Konstantinos Tsioufis, Dimitris Tousoulis

**Affiliations:** 1 Hippokration General Hospital, School of Medicine, National and Kapodistrian University of Athens, 15772 Athens, Greece; vlachakispanag@gmail.com (P.K.V.); spyrsim@gmail.com (S.S.); panos.theofilis@hotmail.com (P.T.); ktsioufis@gmail.com (K.T.); drtousoulis@hotmail.com (D.T.); 2 Endocrine Unit, 2nd Propaedeutic Department of Internal Medicine, School of Medicine, Research Institute and Diabetes Center, Attikon University Hospital, National and Kapodistrian University of Athens, 12641 Athens, Greece; gerogianni.e.maria@gmail.com; 3 Second Department of Cardiology, Aristotle University of Thessaloniki, General Hospital Hippokration, 54942 Thessaloniki, Greece; pakar15@hotmail.com

**Keywords:** tea, polyphenols, cardiovascular health, vascular, blood pressure, blood sugar, lipid profile

## Abstract

Tea consumption is increasingly recognized for its potential benefits to cardiovascular health. This study reviews the available research, concentrating on the major components of tea and their mechanisms of action in the cardiovascular system. Tea is abundant in bioactive compounds, such as flavonoids and polysaccharides, which possess significant antioxidant and anti-inflammatory properties. These compounds play a crucial role in mitigating oxidative stress and inflammation, thereby supporting cardiovascular health. They enhance endothelial function, leading to improved vascular relaxation and reduced arterial stiffness, and exhibit antithrombotic effects. Additionally, regular tea consumption is potentially associated with better regulation of blood pressure, improved cholesterol profiles, and effective blood sugar control. It has been suggested that incorporating tea into daily dietary habits could be a practical strategy for cardiovascular disease prevention and management. Despite the promising evidence, more rigorous clinical trials are needed to establish standardized consumption recommendations and fully understand long-term effects. This review offers a more comprehensive analysis of the current evidence based on endothelium function and identifies the gaps that future research should address.

## 1. Introduction

Tea consumption and especially the flavonoids found in tea have been associated with potential benefits to cardiovascular health. Tea constitutes the main source of flavonoids in many populations. The consumption of 2 g/day of phytosterols and flavonols is recommended [1], with fruits and vegetables being the main sources of flavonols, and catechin—present in tea and cocoa—being the main representative of the cluster [2]. However, the variability in flavonoid concentration within tea, influenced by multifaceted factors including brewing techniques, water temperature, and duration of infusion, poses a significant challenge to comprehending its precise impact on cardiovascular outcomes [3,4]. These factors may also include the concentration of compounds in the tea leaves, the amount of tea leaves utilized to make the infusion, the volume of water employed in its preparation, the duration of brewing, and the level of agitation applied during the infusion process. Additionally, the pH of the water, the thermal treatment, the elapsed time between preparation and consumption, and the amount of tea consumed are also important considerations [4].

The global prevalence of tea consumption as the second most consumed liquid, surpassing even water in some regions, underlines the necessity for a deeper examination of its impact on cardiovascular health [5]. Various mechanisms have been proposed to explain the purportedly beneficial effects of tea on cardiovascular well-being, particularly concerning the intrinsic role of the endothelium and the influence of nitric oxide (NO) in endothelium-dependent vasorelaxation [6,7]. However, the existing studies exhibit considerable heterogeneity not only in the manner of tea consumption (e.g., sweetened or unsweetened) but also in how consumption doses potentially affect the results. It is interesting that the exposures encountered from the use of pure compounds and tea extracts in dietary supplements often entail substantially higher doses, presenting differing efficacy and safety profiles [8].

In this review, we investigate the close relationship between tea consumption and cardiovascular health by studying the role of the endothelium while also acknowledging and addressing the limitations of existing studies. This review focuses on the particular processes by which tea’s principal components affect cardiovascular health, providing a more extensive examination of current data and identifying the gaps that future research should address.

## 2. Tea Types and Chemical Composition

The most popular tea beverages are green tea, black tea, oolong tea, and white tea; made from the infusion of the leaves of *Camellia sinensis* [1]. Tea classification is based on the level of tea leaf fermentation. Green and white tea are minimally fermented during production; oolong tea is partially fermented; while black tea is fermented, thus having a darker color. Tea leaf fermentation determines the antioxidant content in the relevant tea types. In particular, the tea leaves used for green tea production are rapidly heated to a steam or fry heat after harvest, leading to the inactivation of oxidative enzymes and a higher antioxidant content in green tea compared to black and oolong tea [2]. Green tea contains several chemical compounds with antioxidant properties, of which polyphenols have been characterized as the key antioxidant tea components [2].

Polyphenols, flavonoids, and tea polysaccharides are the key bioactive compounds found in tea that contribute to its health benefits [9,10]. Polyphenols are a diverse group of naturally occurring compounds known for their antioxidant properties, which help neutralize free radicals and reduce oxidative stress in the body. Among these, flavonoids are a specific subclass that includes catechins, a major component of tea, particularly green tea. Flavonoids have been extensively studied for their anti-inflammatory, antithrombotic, and vasodilatory effects, all of which play a crucial role in supporting cardiovascular health [9,10]. Tea polysaccharides, another important component, are complex carbohydrates that have garnered attention for their potential hypoglycemic and lipid-lowering effects. These compounds may contribute to better regulation of blood sugar and cholesterol levels, further enhancing the cardiovascular benefits associated with regular tea consumption [9,10]. Together, these bioactive compounds make tea a potent dietary option for promoting overall health, particularly in the prevention and management of cardiovascular diseases. Tea polyphenols, in addition to offering tea its special aroma, have been associated with protection against several diseases, including cardiovascular diseases, malignancies, and neurodegenerative diseases. In green tea, the most abundant polyphenols are catechins, accounting for about 70% of polyphenol content. Green tea catechins include epigallocatechin-3-gallate (EGCG), (2)-epicatechin (EC), (2)-epigallocatechin (EGC), and epicatechin-3-gallate (ECG), of which EGCG is the most abundant and active against reactive species [3]. On the other hand, black tea extracts contain a significantly lower amount of catechins due to the fermentation process that leads to catechin oxidation and polymerization. Thus, thearubigins (large catechin polymers) and theaflavins (catechin oligomers) constitute the predominant polyphenols in black tea [4]. Oolong tea, owing to partial tea leaf fermentation, has an intermediate concentration of catechins (lower than green tea and higher than black tea) and polyphenol oligomers (lower than black tea and higher than green tea). Theasinensins are considered the most significant polyphenols found in oolong tea [2,5].

## 3. Vascular Mechanisms of Action

The beneficial impact of tea and its polyphenols on vascular function has been supported by several studies, most of which highlight the protective effects of tea and its components. Tea’s antioxidant effects, primarily driven by its rich polyphenol content, help neutralize free radicals, reducing oxidative stress in blood vessels [11]. This reduction in oxidative stress preserves the integrity of endothelial cells, which are crucial for maintaining vascular health. By protecting these cells, tea consumption enhances endothelial function, leading to improved vascular relaxation and reduced arterial stiffness [10,11,12]. Additionally, tea’s anti-inflammatory properties help lower the levels of pro-inflammatory markers in the blood, which are often linked to vascular dysfunction. The reduction in inflammation further supports healthy blood vessel function by preventing the buildup of arterial plaque and reducing the risk of atherosclerosis. Together, these effects contribute to an overall improvement in vascular function, promoting better blood flow and reducing the risk of cardiovascular diseases [10,11,12,13].

### 3.1. Antioxidant Effect/Anti-Inflammatory Effect

The antioxidant effects of green tea are highly attributed to catechins, which can act as scavengers of reactive species and metal chelators. Indeed, the presence of the galloyl moiety and phenolic hydroxyl groups determine the antioxidant activity of several tea catechins. These structures donate an electron to reactive oxygen and nitrogen species, leading to free radical reduction. The antioxidant properties of tea products have been strongly correlated with the total polyphenol content and, thus, green tea is a better antioxidant source due to its higher polyphenol concentration compared with black tea [14,15]. Also, a study comparing 30 different tea infusions indicated that green tea generally has the highest antioxidant capacity, compared with black, oolong, white, yellow, and dark teas [16]. The antioxidant effects of green tea and its catechins have been linked to an increase in the total antioxidant capacity (TAC) in murine plasma and myocardium, while white tea increased the TAC only in murine myocardial samples [17,18]. EGCG administration to hens reduced the plasma oxidative biomarker malondialdehyde (MDA), as well as MDA in their eggs, and increased the eggs’ TAC and oxygen radical absorbance capacity. EGCG administration upregulates the expression of mitogen-activated protein kinase (MAPK) and heme oxygenase-1 (HO-1) [19]. In a study including 620 adults aged ≥60 years old, tea, along with coffee, offered the highest increase in dietary TAC and accounted for most of the dietary TAC variability [20]. Catechin hydroxyl groups can also chelate iron and copper ions [21], which may mediate free radical generation.

Besides their direct antioxidant effect, tea catechins also enhance the expression of antioxidant enzymes such as superoxide dismutase, HO-1, and glutathione peroxidase [18,22]. In particular, the upregulation of HO-1 has been linked to the direct genoprotective effects of tea, leading to a significant decrease in lymphocytic DNA damage [23,24]. The antioxidant effects of tea catechins are also mediated by the inhibition of pro-oxidant enzymes, such as nicotinamide adenine dinucleotide phosphate (NADPH) oxidase, xanthine oxidase, and inducible nitric oxide synthase (iNOS). In a murine model, black tea extract reduced NADPH oxidase expression in aortic rings, thereby decreasing reactive oxidative species (ROS) generation [25]. Similarly, green tea polyphenol extracts prevented ROS generation both in vivo in a model of rats on a high-fat diet and in vitro in bovine aortic endothelial cells treated with a high-glucose solution [26]. Specifically, EGCG seemed to reduce the expression of p47 NADPH oxidase subunit in a dose-dependent manner in angiotensin II (Ang-II) activated human umbilical vein cells. However, this effect did not reduce Ang-II-induced ROS generation [27]. Most tea polyphenols, including EGCG, theaflavins, gallic acid, and propyl gallate inhibit xanthine oxidase (XO) [28,29]. XO inhibition may lead to lower uric acid production, as indicated by an experimental study in hepatic cell lines. Unfermented and lightly fermented tea were more potent XO inhibitors than highly fermented black tea [30]. Both EGCG and theaflavin have been associated with a reduction in iNOS mRNA expression in activated murine macrophages. Interestingly, theaflavin and black tea reduced iNOS expression to a greater extent than EGCG [31].

The anti-inflammatory properties of tea have been explored in several experimental and clinical studies. Tea polyphenols decreased lipopolysaccharide (LPS) levels, downregulated expression of the LPS-specific receptor and Toll-like receptor 4, as well as inflammatory biomarkers, such as tumor necrosis factor-alpha, interleukin-1-beta, and interleukin-6, in high-fat diet-induced obese mice [32]. Several tea types, such as green, black, and rooibos, and tea polyphenols, like EGCG and quercetin, have been shown to inhibit microsomal prostaglandin E synthase-1 and, thus, the formation of prostaglandin E2 in activated human monocytes. In particular, green and black tea also reduced cyclooxygenase-2 expression [33]. Oolong tea-derived theasinensins also attenuated COX-2 expression in a dose-dependent manner in activated murine macrophages [34]. The pretreatment of macrophages either with EGCG or theaflavins before activation with Fusobacterium nucleatum abrogated the production of interleukin (IL)-1β, IL-6, tumor necrosis factor-a (TNF-α), and IL8 and matrix metalloproteinases (MMPs) [35]. Additionally, green tea or black tea polyphenol decaffeinated extract in a murine model fed with a high-fat/high-sucrose diet led to a reduction in serum monocyte chemotactic protein-1 (MCP-1) and suppressed MCP-1 gene expression in liver tissue and mesenteric adipose tissue. Oolong tea polyphenol extract reduced only MCP-1 gene expression in mesenteric fat. Adiponectin gene expression was enhanced in the epididymal fat of mice after green and oolong tea polyphenol administration [36]. Furthermore, the consumption of green tea extract for a 5-week period was associated with a reduction in oxidized low-density lipoprotein (oxLDL) levels [37]. Besides oxLDL reduction, EGCG partly inhibits NADPH oxidase leading to lectin-like oxLDL receptor-1 (LOX-1) inhibition in endothelial cells, abrogating intracellular ROS generation and activation of the p38 MAPK pathway. In this way, EGCG is linked to the mitigation of the pro-inflammatory effects of oxLDL [38].

### 3.2. Endothelial Function

The vascular endothelium is acknowledged as an intelligent barrier and a major regulator of blood flow in both microvascular and macrovascular circulation [39]. A reduction in protective substances within the endothelium—such as endothelial nitric oxide synthase (eNOS)—known as endothelial dysfunction, along with high levels of ROS, a key factor leading to this dysfunction, results in an elevated incidence of cardiovascular disease (CVD) [40,41]. Data from animal and human studies have revealed that tea polysaccharides (TPs) have a beneficial effect on endothelial function [42].

In an animal study, the production of reactive oxygen species (ROS) in bovine carotid endothelial cells (BCAECs) was inhibited by tea polyphenols (TPs) through the reduction in NADPH expression. This reduction alleviates angiotensin II-induced hyperpermeability in endothelial cells, potentially preventing CVD [40]. Additionally, research by Liu et al. demonstrated that in BCAECs, TPs activated ERK1/2 while inhibiting p38 MARK signaling in a dose-dependent manner, which led to the downregulation of Caveolin-1 expression, a negative regulator of eNOS, thereby protecting endothelial cells [43]. Furthermore, TPs were found to decrease the expression and secretion of plasminogen activator inhibitor-1 (PAI-1), a critical regulator of atherosclerosis and hypertension, in a time- and dose-dependent manner, contributing to cardiovascular protection [43]. Kim et al. showed that EGCG increased the production of LC3-II and promoted autophagosome formation in primary bovine aortic endothelial cells, resulting in reduced lipid accumulation and improved cardiovascular outcomes [44]. Epicatechin was found to increase nitric oxide (NO) levels in isolated rat mesenteric arteries, which promoted vasodilation via activating iberiotoxin-sensitive K+ channels [45].

Moreover, a study of 20 healthy middle-aged individuals showed that the daily consumption of 200 mL of black tea over 7 days was found to enhance the cutaneous vascular response to gradual local heating up to 42 °C, likely due to the activation of endothelium-derived chemical mediators, such as NO [46]. Additionally, consuming black tea containing 150 mg of polyphenols twice daily for eight days protected blood vessels in hypertensive patients by increasing the number of circulating angiogenic cells and preventing endothelial dysfunction [47]. Green tea catechins, at a dose of 580 mg per day for two weeks, improved endothelial dysfunction in the human forearm and demonstrated an antiatherosclerotic effect in smokers [48]. Finally, green tea treatment was shown to enhance endothelial function in humans as measured by flow-mediated dilation, although this improvement may not be attributable to its isolated compound, EGCG [49].

### 3.3. Antithrombotic Effect

Platelet activation is crucial in CVD, contributing to the pathogenesis of atherosclerosis and the occurrence of acute thrombotic events [50]. Previous studies on the antithrombotic effects of tea have yielded conflicting data. EGCG has been reported to inhibit platelet viability via several mechanisms: it inhibits collagen-mediated phospholipase Cγ2, blocks protein tyrosine phosphorylation, and enhances Ca^2+^-ATPase activity, decreasing platelet aggregation and consequently alleviating atherothrombosis [51]. In vitro studies have shown that green tea catechins mainly impact the platelet aggregation assays inducing ADP, collagen, epinephrine, and the calcium ionophore A23187, rather than affecting the anticoagulation process [52,53]. EGCG has also been demonstrated to induce tyrosine phosphorylation of platelet-associated proteins, such as Syk and SLP-76, and to lower the phosphorylation levels of focal adhesion kinases, therefore, enhancing platelet aggregation [54,55]. Kang et al. discovered that catechol influences intracellular calcium levels in platelets by activating Ca^2+^-ATPase and inhibiting the production of inositol trisphosphate. This sequence of events subsequently hinders fibrinogen binding to GPIIb/IIIb receptors, thereby reducing platelet aggregation [56]. Endothelial cell injury also triggers inflammatory and oxidative responses, which are crucial contributors to thrombosis [39]. A recent study found that the combination of EGCG and warfarin significantly decreased thrombus weight in a rat model of deep vein thrombosis [57]. Further in vitro experiments showed that this combination protected human umbilical vein endothelial cells (HUVECs) from oxidative stress and prevented apoptosis. The protective effect was linked to the inhibition of HIF-1α-mediated activation of the PI3K/AKT and ERK1/2 signaling pathways [57].

**Figure 1:**
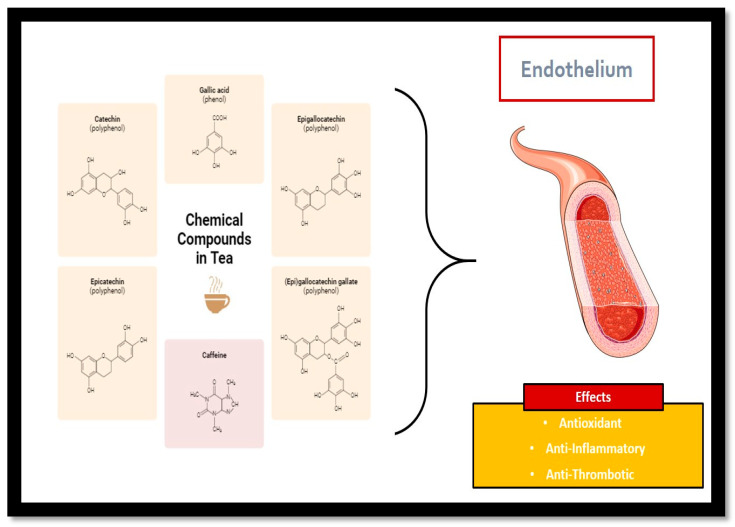
Chemical components of tea and their influence on the endothelium.

Despite in vitro studies suggesting a possible effect of tea consumption on platelet aggregation, a limited number of human trials have failed to confirm this correlation. Specifically, Duffy et al. demonstrated that, among patients with coronary artery disease (CAD), the consumption of black tea, despite the presence of antioxidant flavonoids known to decrease platelet aggregation in vitro, did not lead to altered dose-dependent platelet aggregation in response to Adenosine diphosphate (ADP) and thrombin receptor-activating peptide either acutely or chronically [58]. Furthermore, in a randomized controlled crossover study involving twenty-two individuals, the consumption of 5 cups of black tea per day for 4 weeks, compared with hot water, resulted in increased 24 h urinary excretion of 4-O-methylgallic acid and lower soluble P-selectin levels, but did not affect other adhesion molecules, platelet aggregation in response to collagen or ADP, or coagulation and fibrinolytic factors [59]. In a subsequent study by the same investigators, twenty healthy participants showed that while black tea significantly increased urinary 4-O-methygallic acid concentrations, it did not inhibit postprandial platelet aggregation induced by collagen or ADP, suggesting that reduced postprandial platelet aggregability does not contribute to the cardiovascular benefits of black tea [60] (Figure 1).

## 4. Cardioprotective Effect

Numerous animal and in vitro studies have demonstrated that tea diet supplementation benefits cardiovascular health through its pleiotropic effects. Regular tea consumption on top of the standard-of-care medication seems to decrease all-cause and CV mortality rates via the slight regulation of the main cardiovascular risk factors [61,62,63]. The focus of our review will be green tea which is the most well-studied tea supplementation to date.

### 4.1. Blood Pressure Regulation

Hypertension is one of the leading CVD risk factors remaining a persistent global health concern, contributing to 10.8 million deaths worldwide [64]. The reports on the effect of tea consumption on systolic (SBP) and diastolic blood pressure (DBP) are inconsistent. Several meta-analyses have sought to investigate the relationship between tea consumption and blood pressure regulation, with varying degrees of success. In 2014, Onakpoya et al. conducted a meta-analysis that demonstrated a significant reduction in systolic blood pressure (SBP) among individuals consuming green tea, though no significant effect was observed on diastolic blood pressure (DBP). This study highlighted the potential of green tea to impact cardiovascular health, particularly through its effects on SBP [65]. Subsequent research has supported these findings, with two studies reporting that even short-term tea supplementation can lead to notable decreases in both SBP and DBP [7,66]. These effects appear to be more pronounced in individuals with established hypertension. For instance, a study focusing on hypertensive patients found that green tea consumption resulted in significant reductions in both SBP and DBP [7,66]. This finding aligns with a comprehensive umbrella review conducted in 2022, which consolidated evidence from various studies and confirmed the beneficial impact of green tea on blood pressure among hypertensive individuals [67]. The mechanisms underlying these blood pressure-lowering effects are thought to be multifaceted. Green tea is rich in catechins, such as epigallocatechin gallate (EGCG), which have been shown to exert vasodilatory effects, improve endothelial function, and inhibit the activity of angiotensin-converting enzyme (ACE). These actions collectively contribute to the reduction in vascular resistance and, consequently, lower blood pressure [4]. Moreover, the antioxidant properties of green tea catechins help mitigate oxidative stress, which is a known contributor to hypertension. More specifically, researchers assume that the reduction in reactive oxygen and nitrogen species enhances the function of antioxidant enzymes (catalase, superoxide dismutase), protecting against endothelial dysfunction and consequently regulating blood pressure. Another potential mechanism is the upregulation of circulating adiponectin, which is linked to blood pressure reduction due to its antiatherogenic properties, enhancement of insulin sensitivity, and reversal of salt-induced hypertension. Green tea’s influence on other metabolic parameters, such as weight reduction and improved lipid profiles, may indirectly contribute to its antihypertensive effects [4,12].

### 4.2. Cholesterol Management

The beneficial effects of tea consumption, particularly green tea, have been demonstrated in numerous studies with no debatable results between them. Regular consumption has been shown to significantly reduce total and low-density cholesterol levels. A recent meta-analysis revealed a significant decrease in triglyceride levels with supplementation for more than one year, along with an increase in high-density cholesterol levels [68]. Several mechanisms have been proposed to explain the beneficial effects of tea consumption on lipid profile. The consumption of flavonoids and especially catechins, which are in high concentrations in tea leaves, present a strong antioxidant effect [69]. Catechins, particularly the EGCG found predominantly in green tea, play a crucial role in inhibiting the oxidation of lipoproteins, with a specific LDL oxidation. OxLDL is a major contributor to the progression of atherosclerosis, making catechins’ antioxidative properties particularly beneficial for cardiovascular health. Moreover, the flavonoids present in green tea exert significant hypolipidemic effects by reducing micellar solubility and the intestinal absorption of cholesterol [69,70]. This process subsequently results in lower hepatic cholesterol concentrations, contributing to overall lipid regulation. The hypolipidemic properties of green tea are further linked to its weight-reduction capabilities. Numerous studies have demonstrated that regular green tea consumption results in modest but statistically significant weight loss [71]. This weight reduction may further enhance the lipid-lowering effects of green tea, as body weight is closely related to lipid metabolism. Collectively, the antioxidative and lipid-regulating properties of the catechins and flavonoids in green tea underscore its potential as a dietary intervention for reducing the risk of atherosclerosis and managing hyperlipidemia. These findings support the potential inclusion of green tea in dietary suggestions aimed at improving cardiovascular health and metabolic outcomes [69,70,71].

### 4.3. Blood Sugar Control

**Figure 2:**
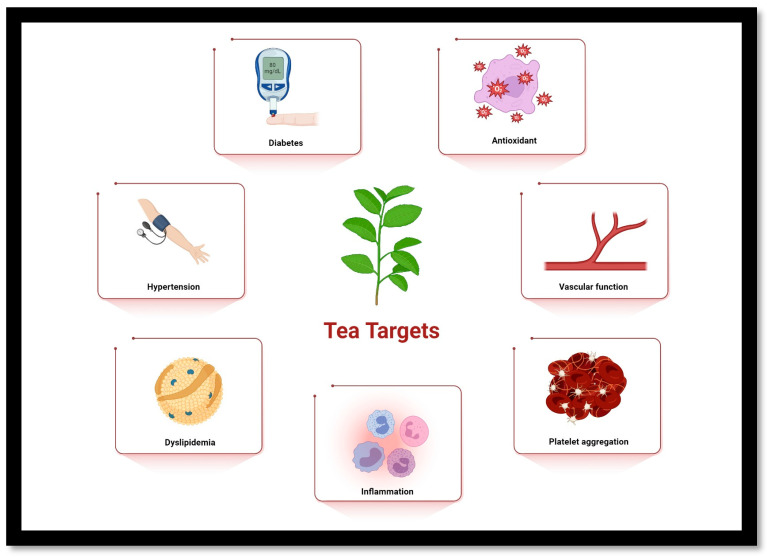
Tea targets in cardiovascular health. Created with https://www.biorender.com/.

The data are scarce and debatable concerning the association between tea consumption and blood sugar control. Several large studies have investigated the relationship between tea consumption and the risk of developing diabetes mellitus, yielding mixed results [72,73]. While some studies have reported a significant reduction in diabetes risk associated with tea consumption, others have found neutral effects. Notably, two large-scale studies provide compelling evidence supporting the beneficial impact of tea on diabetes mellitus and overall mortality among diabetic patients. The first study, encompassing a cohort of approximately 0.5 million individuals, demonstrated that regular tea consumption was associated with a reduced prevalence of diabetes mellitus [74]. Similarly, another substantial study involving 27,000 participants found that tea drinkers not only had a lower prevalence of diabetes but also experienced a decreased risk of all-cause mortality [75]. This suggests that tea consumption may confer protective benefits beyond glycemic control, potentially improving overall survival rates among those with diabetes [76,77]. However, the effects of green tea on other glycemic indices, such as fasting blood glucose and hemoglobin A1c (HbA1c) levels, remain contentious. While some studies report significant improvements in these indices, others find no notable effects [78,79,80]. This disparity may be attributed to differences in study design, populations, and the amounts and types of tea consumed. For instance, variations in the bioactive compounds present in different tea types, the duration of tea consumption, and participants’ baseline health statuses could all influence outcomes. Additionally, the interaction of tea with other dietary and lifestyle factors may contribute to the inconsistency in the findings. Although previous meta-analyses did not demonstrate significant effects on fasting insulin, HbA1c levels, or the Homeostatic Model Assessment for Insulin Resistance (HOMA-IR) [79], recent studies have provided substantial evidence indicating significant reductions in fasting blood sugar and HbA1c levels [78,80]. This suggests that, while earlier research may have overlooked some benefits, more recent investigations highlight the potential for specific interventions to improve glycemic control and offer new insights into their metabolic impacts (Figure 2).

## 5. Clinical Implications—Conclusions

Natural compounds derived from food and plants have garnered significant interest due to their perceived low toxicity, cost-effectiveness, and widespread availability. Nonetheless, their precise physiological mechanisms, particularly concerning the cardiovascular system, remain incompletely elucidated. CVD is a complex condition influenced by numerous factors wherein tea components exhibit diverse effects across various processes: antihypertensive, lipid-lowering, antioxidant, anti-inflammatory, antiproliferative, antiangiogenic, antiatherosclerotic, endothelial function restoration, antithrombotic, and myocardial protective effects [81,82].

Despite data from epidemiological studies and meta-analyses indicating that tea may offer protective effects against CVD, translating these findings into specific dietary recommendations presents significant challenges. Notably, there are no randomized clinical outcome trials evaluating tea consumption and epidemiological studies are susceptible to confounding factors inherent in the lifestyles of tea drinkers and non-drinkers. Unlike drug trials, conducting randomized clinical trials with tea is complicated by ethical concerns and the ubiquitous nature of tea consumption in daily diets. Additionally, uncertainties persist regarding the type, dosage, and specific populations for study. The cost of conducting large-scale clinical outcome studies further complicates matters, especially given the absence of profit-driven sponsors for whole foods like tea.

Addressing these complexities necessitates further animal experimentation, large-scale cohort studies, and human randomized trials. In conclusion, accumulating evidence underscores the pivotal role of TPs in preventing and treating CVD by modulating various cellular signaling pathways. Nonetheless, comprehensive investigations into the precise molecular mechanisms of TPs across diverse cell types are imperative for a deeper understanding of their therapeutic potential and to alter the “business as usual” paradigm, thereby advancing our mission to reduce the burden of CVD.

## Data Availability

Upon request to the authors M.S.
